# Time to assess more than prognosis: advancements and challenges in transcatheter tricuspid valve interventions

**DOI:** 10.3389/fcvm.2024.1447411

**Published:** 2024-08-09

**Authors:** Felix Rudolph, Maria Ivannikova, Tanja K. Rudolph, Volker Rudolph, Muhammed Gerçek, Kai P. Friedrichs

**Affiliations:** Clinic for General and Interventional Cardiology/Angiology, Herz- und Diabeteszentrum NRW, Ruhr-Universität Bochum, Med. Fakultät OWL (Universität Bielefeld), Bad Oeynhausen, Germany

**Keywords:** TTVI, quality of life, health status, RV function, RV remodeling

## Abstract

We provide an overview about the current landscape of transcatheter tricuspid valve interventions (TTVI) and summarize recent findings from trials including TRILUMINATE, TRILUMINATE Pivotal, bRIGHT, TRICLASP, TRISCEND, TRISCEND II, TRICUS, and Cardioband TR EFS. These studies have demonstrated the safety and efficacy of TTVI. Yet, they have failed to show a prognostic benefit over conservative treatment. On the other hand, significant improvements in health status assessments have been observed. Assessment of right ventricular (RV) function prior to tricuspid interventions is crucial, as changes in preload and afterload may lead to RV failure which is associated with a high mortality. Therefore, this review emphasizes the impact of TTVIs on quality of life and explores the influence of RV dysfunction on therapeutic success and prognosis.

## Key points

(1) Transcatheter tricuspid valve interventions (TTVIs) are safe and efficient.
(2) While currently no prognostic benefit is evident, TTVIs are associated with an improvement in health status.
(3) Early evaluation and treatment of TR result in improved outcomes.
(4) Assessment of RV dysfunction remains difficult and is associated with impaired outcome.

## 1 Introduction

Relevant tricuspid regurgitation (TR) is highly prevalent among elderly patients, affecting approximately 4% in this population (1, 2). It is associated with a significant symptomatic burden, leading to a high rate of morbidity, hospitalization, and mortality. Long-term survival rates at eight and ten years for conservatively managed moderate TR range from 22% to 26%, with TR severity and progression being independent predictors of survival (3, 4). TR is classified into primary (or degenerative), secondary (or functional), and cardiac implantable electronic device (CIED)-related forms, depending on the underlying pathological mechanism. Primary TR, accounting for a small fraction of cases, arises from degenerative alterations of the tricuspid valve apparatus itself, such as those caused by infective endocarditis, rheumatic heart disease, endomyocardial fibrosis, or iatrogenic damage. However, over 90% of cases are secondary, often due to left-sided cardiac pathologies, resulting in enlargement of the right ventricle (RV) or right atrium (RA), which leads to pressure and/or volume overload and subsequent progressive RV remodeling. Accordingly, secondary TR can be further subdivided into atrial and ventricular forms (1, 5). In recent years the awareness of TR has shift TR from a surrogate marker of advanced cardiac disease to an independent entity with significant prognostic relevance.

This paradigm shift has steered treatment away from solely conservative measures towards more interventional and preventative approaches (6, 7).

The European Guidelines recommend surgical repair or replacement for symptomatic primary TR without severe right ventricular dysfunction. This intervention is also advised for cases of secondary TR during left-sided surgeries. The American Guidelines recommend surgery only at the time of left-sided surgery (8). Isolated surgical correction of secondary TR is currently not recommended in either of the guidelines due to insufficient evidence of clinical benefit (1, 8). Conservative management primarily relies on oral diuretics to balance the fluid status, a strategy that can be challenging in a home setting and may lead to progressive clinical deterioration and recurrent hospitalizations for intravenous diuretic therapy (1, 9). As a promising alternative, transcatheter tricuspid valve interventions (TTVI) have emerged, expanding treatment options for patients at high surgical risk. Although recent data indicate that these interventions might not provide a prognostic benefit, they have been shown at least to improve patients’ quality of life (9).

In this review, we explore current transcatheter treatment options for TR and their impact on patients’ quality of life. Additionally, we highlight the role of RV function on therapeutic success and prognosis.

## 2 What are the treatment options?

Interventional procedures for treating tricuspid valve insufficiency can be categorized into those that repair the valve and those that replace it. The most experience has been gained with the transcatheter edge-to-edge (T-TEER) approach, where clips are used to bring the leaflets together, thereby reducing insufficiency. Another repair approach is percutaneous direct annuloplasty using the Cardioband system (Edwards Lifesciences), where anchors are inserted into the annulus. A band connected to these anchors can subsequently be tightened, resulting in a reduction of the annulus size and consequently, the insufficiency (10). For valve replacement, the EVOQUE system (Edwards Lifesciences) is approved (11, 12). Another system is the LuX-Valve System, which has so far been implanted via a right-sided mini-thoracotomy. However, initial experiences are also being gathered with a new generation that can be implanted through the jugular vein (13–15). Some patients may not be suitable for either of these procedures. In these rare cases, a heterotopic valve replacement using the TricValve System may be considered, where a prosthesis is implanted in both the superior and inferior vena cava to address systemic reflux (16).

While available data for conservatively treated TR indicate poor long-term survival rates under medical therapy alone, the strategy for medical heart failure treatment has been significantly refined in recent years, particularly with the introduction of sodium-glucose cotransporter 2 inhibitors (SGLT2i). Current guidelines recommend a four-pillar medical approach for patients with heart failure with reduced ejection fraction (HFrEF). These pillars are an angiotensin-converting enzyme inhibitor (ACEi) or an angiotensin receptor blocker (ARB) or an angiotensin receptor-neprilysin inhibitor (ARNi), a β-blocker, a mineralocorticoid receptor antagonist (MRA), and an SGLT2i (17). The latter have been proven to reduce the combined risk of worsening heart failure or cardiovascular death in HFrEF and HFpEF patients in randomized controlled trials (18, 19). Additionally, rehospitalization rates were shown to decrease with the increasing number of medical pillars prescribed for HFrEF patients (17). While these advances are groundbreaking, some limitations apply, particularly hypotension and impaired renal function, which may limit the applicability of all four pillars.

## 3 How can we quantify therapeutic success?

Common metrics used to assess functional status include the NYHA functional class, the six-minute walking distance (6-MWD), the rate of rehospitalization, and the Kansas City Cardiomyopathy Questionnaire (KCCQ) score. The KCCQ score combines and quantifies five domains: physical limitation, quality of life, social aspects, self-sufficiency, and clinical symptoms. It is scored on a scale ranging from 0 to 100, with higher scores indicating a better health status. Changes in the KCCQ score of 5–10 points represent small clinical changes, 10–20 points indicate moderate changes, and changes of 20 points or more are considered large clinical changes (20, 21).

In the context of TR, procedural success is typically measured by the reduction in TR grade. However, the underlying pathological mechanisms of TR are complex and closely connected to right heart function. In conditions such as pulmonary hypertension, which impose a chronic burden on the right heart, it is well established that the RV can undergo significant reverse remodeling if the underlying cause is effectively addressed. Common echocardiographic assessments regarding RV function and cardiac remodeling include tricuspid annular systolic excursion (TAPSE) and RV basal diameter, favored for their ease of use and wide availability.

For a more comprehensive evaluation, advanced echocardiographic parameters can be employed. These include the RV-PA ratio [relating TAPSE to right ventricular systolic pressure (RVSP)], fractional area change (FAC), and RV free-wall longitudinal strain (5). Additionally, the systemic impact of TR can be estimated by measuring the diameter of the vena cava inferior and observing hepatic vein backflow.

Notably, accurately assessing QoL outcomes is challenging in unblinded procedure trials. The lack of blinding may introduce significant placebo and nocebo effects, where patients’ perceptions of their health can be unduly influenced by their knowledge of receiving or being denied a procedure. This bias can notably affect QoL measures and, to a lesser extent, metrics such as the six-minute walking distance. As we move forward, a more critical approach to study design, including the use of blinded assessments, appropriate control groups, and the assessment of hard endpoints, will be vital to ensure the reliability and validity of our findings.

## 4 What new data were presented?

New three-year follow-up data from the TRILUMINATE study were presented by Georg Nickenig at PCR London Valves 2023, investigating the safety and efficacy of the TriClip system. This data demonstrated a robust decrease in TR grade to moderate or less in 38 of 48 patients with available data (79%). Of 98 patients included, 18 (18%) had died from cardiovascular causes. For a paired group of 54 patients with available baseline and follow-up data, an improvement in NYHA class was observed. At baseline, 24% of patients were classified as NYHA class II or better, whereas this number increased to 82% at follow-up. Furthermore, 50% of patients achieved an increase in KCCQ score by 10 or more points. The right ventricular end-diastolic basal diameter (RVEDD) decreased from an initial 5.3 cm to 4.8 cm after one year, maintaining a slight increase to 4.9 cm after three years. Additionally, the tricuspid annular diameter decreased from 4.3 cm to 4.1 cm (22).

The findings of the TRILUMINATE study are limited to the first generation of the TriClip device. By contrast, the post-market bRIGHT study, which included newer generations of the TriClip was presented by Rodrigo Estevez-Loureiro at the same conference, seems to confirm these findings: for 92 patients with available data at baseline and follow-up, 67 (73%) achieved a TR grade of moderate or less. NYHA class was available in 112 patients, with 77 (69%) classified as NYHA class II or better at follow-up (23).

Kodali et al. presented one-year follow-up data from the TriCLASP study at PCR London Valves 2023, assessing the safety and efficacy of the PASCAL system for treating TR. Among the 119 patients included, 106 (86%) achieved a TR grade of moderate or less, a notable increase compared to the 30-day data. Improvement was also seen in the NYHA class, with 103 (92%) patients improving to class II or better. The KCCQ score improved by 19 points on average, and the 6-MWD increased by 103 meters. A significant reduction in hospitalizations for cardiac decompensation was observed, decreasing by 56.4% compared to the year prior to intervention. Significant changes were also noted in the RV mid diameter (decreasing from 4 cm to 3.5 cm) and the RA volume (from 148.9 ml to 130.6 ml), although no significant changes were observed in the FAC and TAPSE (24).

As most patients present secondary TR, data on the feasibility of T-TEER in primary TR are scarce. Dannenberg et al. published data comparing procedural T-TEER results between patients treated for primary and secondary TR. They included 339 patients, of whom 44 (13%) were classified as primary TR. In this primary group, a TR grade of moderate or less was achieved in 33 (76%) patients. The rates of complications and postprocedural events were comparable to those of the secondary group and did not differ from other recent trials (25).

The TRILUMINATE Pivotal trial was the first randomized controlled study to compare transcatheter intervention (specifically T-TEER) with optimal medical therapy (OMT). With T-TEER, a robust reduction in TR grade was observed, with 158 (98.3%) patients free of adverse events after 30 days. This trial, which included 350 patients randomly assigned in a 1:1 ratio, achieved its primary endpoint, a composite including all-cause mortality, tricuspid-valve surgery, hospitalization for heart failure, and improvement of quality of life as measured by at least a 15 point-increase in the KCCQ score. The win ratio was 1.48 in favor of T-TEER. While mortality and rehospitalization rates did not differ after one year, the main benefit was an improvement in quality of life. After 30 days, 140 (87%) T-TEER patients were classified with a TR grade of moderate or less, compared to only 7 (4.8%) of the OMT patients. A sub-analysis comparing the health statuses of T-TEER and OMT patients revealed a significant increase in the number of T-TEER patients who were alive and well (defined as a KCCQ of 60 or higher without a decrease of more than 10 points at one year), with 120 (74.8%) T-TEER patients meeting these criteria compared to 67 (45.9%) OMT patients. This analysis also indicated that patients with worse health statuses at baseline were more likely to benefit from the intervention. Although the TILUMINATE Pivotal trial did not demonstrate a prognostic benefit, it did show significant improvements in quality of life and health status (9, 26).

The TRISCEND study, also presented at PCR London Valves 2023 by Raj Makkar, investigates the safety and efficacy of the EVOQUE prothesis for TTVR. Two-year follow-up data was achievable for 90 of the initially 208 treated patients. Technical success was achieved in 197 (94.7%) of all patients, with 23 of 141 (16.3%) having died from cardiovascular causes after two years. Bleeding events occurred in 43 (30.5%) patients. The rate of hospitalization for cardiac decompensation was reduced by 57% compared to the pre-procedural year. TR was eliminated in 65 of 66 patients (98.5%) with available data, as they were classified with a TR grade of mild or less after two years. Subsequently, the rate of NYHA II or better improved from 26.9% at baseline to 86.5% after two years. This trend was also observed in the KCCQ score, which improved by 20 points, and the 6-MWD, which improved by 46.7 meters. Significant changes were observed in the RVEDD, which decreased from 41.8 mm to 31.6 mm, TAPSE (from 16.5 mm to 14.1 mm) and FAC (from 40.6% to 32.3%) (11).

The subsequent TRISCEND II study is a randomized controlled trial comparing the safety and efficacy of the EVOQUE system in combination with OMT to OMT alone in a 2:1 ratio design. In total, 400 patients were enrolled, with follow-up currently ongoing. Early data from the first 150 patients who completed follow-up until the first safety endpoint after six months were presented at TCT 2023 and London Valves 2023. After six months, a TR grade of moderate or less was achieved in 80 of 82 (98.8%) patients in the intervention group (EVOQUE + OMT), compared to only 8 of 37 (21.6%) in the OMT alone group. A win ratio was calculated, combining the quality-of-life scores KCCQ, NYHA and 6-MWD. This showed significant superiority in the treatment group of a win ratio of 4.6. Additionally, the major adverse event rate was lower compared to the expected rate derived from historical data on isolated tricuspid replacement surgery. Compared to the initial TRISCEND study, the rate of severe bleeding events was lower at 10.5% (vs. 30.5%). The need for pacemaker implantation was reported in 14.7% of cases (12).

Early experience data from the first generation of the LuX-Valve system, implanted via a right-sided mini-thoracotomy in 126 Chinese patients, was presented at PCR London Valves 2023. For interventional TR treatment, these patients were relatively young, averaging 65.8 years of age. Device and procedural success were achieved in 123 (98%) cases. Three patients (2.4%) died in hospital. After one year, the all-cause mortality was 10%. Major adverse events occurred in 23.8% of cases after one year, the most common being right atrial or thoracic bleeding at 12.7%. Only 1.6% of patients experienced a new onset of total atrioventricular blockade necessitating pacemaker implantation. A TR grade of moderate or less was achieved in 94.4% of 107 cases with available follow-up data. The NYHA class improved from all patients being classified as III or IV to 79.8% being classified as II or better, but for 27 patients no NYHA class at follow-up was available. The 6-MWD improved by 42 meters (15). A CE-mark trial for the second generation of the device, providing a transjugular access, is currently ongoing, but the first experience in ten patients were recently published, proving the feasibility of the transjugular delivery system (13).

First one-year follow-up data for the bicaval implanted TricValve protheses from 44 patients in the TRICUS registry showed clinical improvement in 42 (95.5%) patients, as measured by at least a 15-point increase in KCCQ score, improvement in NYHA class to I or II, or an increase of at least 40 meters in the 6-MWD. Abolishment of hepatic vein backflow was achieved in 28 (63.8%) patients, with consequent reduction in rates of congestive symptoms, doses of oral diuretics, and levels of NT-proBNP. All-cause mortality was 6.8% (27).

One-year follow-up data after Cardioband implantation, originating from an early feasibility study and published in 2022, reported a residual TR grade of moderate or less in 19 (73%) patients, with a KCCQ score improvement by 19 points after one year (10). Recent data comparing T-TEER to TTAR in 161 patients found similar technical success and periprocedural safety between the techniques. However, TTAR patients had more severe TR and a greater coaptation gap at baseline. A propensity score-matched analysis revealed greater efficacy of TTAR in achieving optimal results of TR grade of moderate or less. Nonetheless, procedural time and bleeding events were higher in the TTAR group, underscoring the importance of adequate patient selection (28). An updated new generation of the device and subsequent further data are expected in the future. These recent findings are summarized in Table 1.

**Table 1:** Summary of recent TTVI trials.

Trial	Triluminate		Triluminate Pivotal		BRIGHT		TriCLASP		Triscend		Triscend II		LuX-Valve pivotal		TRICUS		Cardioband TR EFS	
Modality	T-TEER								TTVR						TBVI		TTAR	
PMID	Congress proceeding		36,876,753		Congress proceeding		37,137,586		Congress proceeding		Congress proceeding		Congress proceeding		38,069,986		36,202,561	
Year	2023		2023		2023		2023		2023		2023		2023		2024		2022	
*n*	98		350		200		65		208		150		126		44		37	
Data	BL	3yFU	BL	1yFU	BL	2yFU	BL	1yFU	BL	2yFU	BL	6mFU	BL	1yFU	BL	1yFU	BL	1yFU
TR grade ≤ moderate	5%	79%	2.3%	88.1%	1.6%	73%	3%	86%	13%	100%	0%	98.8%	0%	99.1%	0%	0%	0%	73%
NYHA class ≤ II	24%	82%	43%	83.9%	19%	69%	29.2%	92%	26.9%	86.5%	20.8%	90%	0%	79.8%	0%	62.2%	35.1%	92.3%
6-MWD	na		na		na		208	311	207	258	247	257.6	324.3	383.2	253.4	284	na	
KCCQ score	Increase ≥10 pts. in 50% of patients		55.1	67.4	43.1	na	53	72	45.9	71.7	50.7	72.2	na		36.5	65.6	57.3	76.4

In the controlled trials, data are derived from the intervention arms. 6-MWD, six-minute walking distance (in meters); 6mFU, follow-up after six months; 1yFU, follow-up after one year; 2yFU, follow-up after two years; 3yFU, follow-up after three years; BL, baseline; KCCQ, Kansas city cardiomyopathy questionnaire; na, not available; n, number of patients (enrolled); NYHA, New York Heart Association; PMID, PubMed-ID; pts., points; TBVI, transcatheter bicaval valves implantation; T-TEER, transcatheter edge-to-edge repair; TR, tricuspid regurgitation; TTAR, transcatheter tricuspid annular reduction; TTVI, transcatheter tricuspid valve interventions; TTVR, transcatheter tricuspid valve replacement.

## 5 What are the implications for clinical practice?

### 5.1 When is the optimal time for intervention?

Based on eight clinical and echocardiographic parameters (age ≥70 years, NYHA class III or IV, signs of right-sided heart failure, daily dose of furosemide ≥125 mg, glomerular filtration rate ≤30 ml/min, elevated total bilirubin, impaired left ventricular function, and moderate or severe right ventricular dysfunction), the TRI-SCORE was created and evaluated to predict in-hospital mortality following isolated tricuspid valve surgery. The maximum possible score is 12, associated with the highest rate of predicted in-hospital mortality at 65% (29). This score was applied to the TRIGISTRY cohort, comprising a total of 2,413 patients with severe isolated functional TR and available data on two-year survival (1,217 managed conservatively, 551 underwent isolated surgical repair, and 645 transcatheter valve repair). This revealed two key finding: (1) survival was significantly worse in patients with a high TRI-SCORE (≥6), indicating patients with advanced disease, and (2) survival was significantly worse in patients who underwent unsuccessful transcatheter valve repair, defined as TR-grade worse than moderate. These findings underscore the importance of early diagnosis and treatment of TR (30).

### 5.2 Which patients do we help, and which do we harm?

Patients in the TRIGISTRY registry with a low TRI-SCORE (≤3) demonstrated better survival rates following surgical or successful transcatheter repair. This beneficial effect was also observed in patients at intermediate risk (TRI-SCORE 4–5), although it was less pronounced. Importantly, within the intermediate and high-risk groups, survival rates were better among those treated conservatively than those who underwent unsuccessful transcatheter repair. In the low-risk group, the survival rates of conservative and unsuccessful interventional treatment were similar (30). This suggests that certain patients may be at risk of harm from interventions, particularly those with complex anatomies where an effective reduction of TR may not be anticipated. Additionally, a TRI-SCORE ≥ 8 was associated with an increased risk of adverse clinical events (30). Recently, the GLIDE Score was proposed, comprising five anatomical features of the tricuspid valve in a Score from 0 to 5. These parameters are size of septolateral gap exceeding 6 mm, the predominant jet location, image quality, chordal structures density and en-face TR jet morphology. Patients with a GLIDE Score of 4–5 showed significantly worse immediate TR reduction following T-TEER (31). If this score is capable to predict long-term TR reduction and functional benefit remains to be investigated. Apart from sizeable parameters such as echocardiographic data, laboratory results, and survival rates, the benefit of a intervention can also be assessed by an improvement in symptoms. Although the TRILUMINATE Pivotal trial, which compared T-TEER to OMT, did not demonstrate a prognostic benefit regarding death or heart failure hospitalization at one-year follow-up, it did reveal symptomatic relief. Suzanne Arnold conducted a sub-analysis of the TRILUMINATE Pivotal patients, confirming that T-TEER resulted in substantial symptomatic and functional benefits. Notably, patients with a preserved cardiac index of 2 L/min/m^2^ or greater reported a greater increase in health status compared to those with a depressed cardiac index. Since most included patients had preserved left-ventricular function, the decrease in cardiac index was primarily attributed to impaired RV function. Additionally, patients with a low KCCQ score at baseline were more likely to benefit from T-TEER. This suggests that the ideal patient population for T-TEER comprises those with a high symptomatic burden but preserved RV function (26). However, the applicability of the TRILUMINATE trial findings to real-world conditions may be limited. Lukas Stolz applied the TRILUMINATE eligibility criteria to a registry of 962 real-world patients and found that only 527 (55%) met these criteria. When comparing those eligible and ineligible for TRILUMINATE, both groups exhibited comparable improvements in TR grade, NYHA class, and 6-MWD following T-TEER. However, survival rates were significantly lower among the ineligible patients, who also presented more comorbidities. Consequently, it remains uncertain whether these patients could benefit from T-TEER not just in terms of survival rates, but also in terms of quality of life and of rehospitalization (32). While generally the presented follow-up data indicated improvements in functional status, these insights might be influenced by survivorship bias. The overarching goal should always be patient-oriented. Technical success is only useful if it is also reflected in an improvement in quality of life or life expectancy. According to current findings, transcatheter tricuspid valve interventions appear to positively influence quality of life. Moreover, it is conceivable that treating the systematic effects of chronic reflux could also have further positive effects, such as an increase in heart failure medication dosage with its proven positive impacts. Appropriate preoperative and postoperative care is essential. Telemedicine, which includes frequent weight monitoring and data transmission to a research center, could serve as an additional cornerstone, playing a pivotal role in the early detection of cardiac decompensation before and after tricuspid valve interventions, thereby further optimizing treatment outcomes.

### 5.3 What are RV remodeling and reverse remodeling?

Chronic burden on the RV due to various underlying disease mechanisms, such as pulmonary hypertension, left-sided cardiac disease, myocardial injury, TR, leads to structural and functional changes. These adaptations may involve alterations in RV dimensions, geometry, wall hypertrophy, and impairment of functional parameters such as TAPSE and FAC. Within the RV myocardium, this can result in hypertrophy, dilation, and fibrosis. Initially, the RV may adapt to these conditions through adaptive remodeling. However, following prolonged chronic burden, it may decompensate, leading to maladaptive remodeling. If the underlying cause of the remodeling, in this case, TR, is effectively addressed, the RV is capable of an impressive reversal of these changes, a process known as reverse remodeling. In clinical practice, this may manifest as an increased TAPSE or decrease in RV dimensions following tricuspid valve interventions.

### 5.4 Physiological implications of TR reduction on RV function

**Figure 1:**
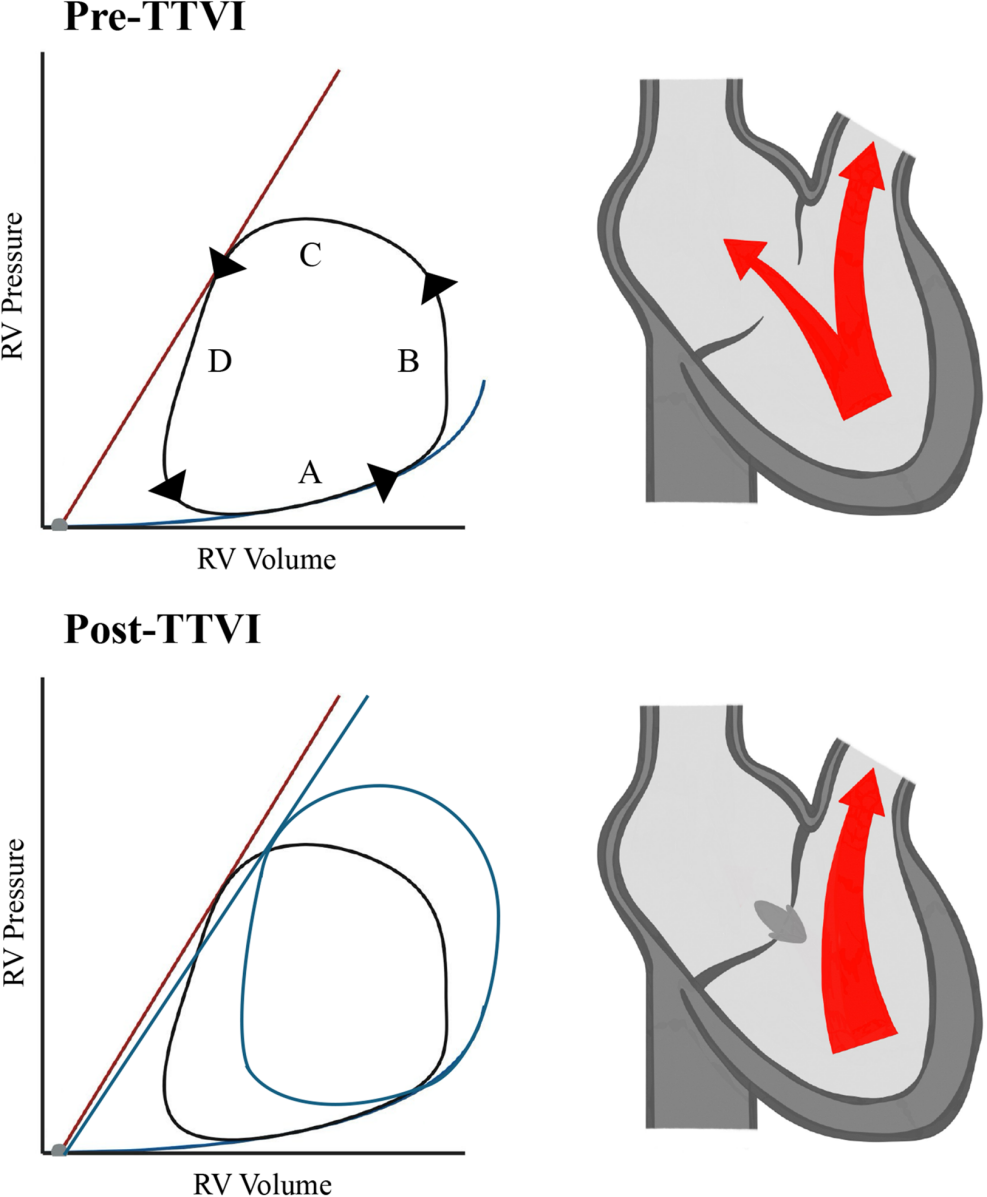
RV pressure/volume relations. A = filling phase, B = isovolumetric contraction, C = ejection phase, D = isovolumetric relaxation. RV, right ventricle; TTVI, transcatheter tricuspid valve intervention.

All TR interventions lead to changes in the RV preload, afterload, and consequently, RV function. The RV cardiac cycle includes four phases: filling, isovolumetric contraction, ejection, and isovolumetric relaxation (Figure 1). In TR patients, the RV experiences relief from increased afterload due to the reflux, typically resulting in higher RV ejection fractions, a phenomenon known as the “pop-off” effect. Eliminating TR decreases preload and increases afterload, as blood volume shifts from the low-pressure zones of the RA to the relatively high-pressure pulmonary arteries (PA). This increase in afterload elevates peak RV and PA pressures and cardiac work, while reducing stroke volume and ejection fraction, which ultimately may lead to decreased RV-PA coupling. The RV is particularly sensitive to increases in afterload. Therefore, patients with pre-existing impaired RV function may be at risk of acute RV failure. This underscores the critical importance of thorough assessment of RV function prior to tricuspid valve interventions (33, 34). Given these theoretical implications, an analysis of patients from the TriValve registry who underwent transcatheter tricuspid valve interventions revealed that low-cardiac output syndrome was rare but associated with increased preprocedural morbidity, advanced biventricular dysfunction, and periprocedural adverse events, rather than stemming directly from acute RV failure. In conclusion, the role of RV dysfunction and RV failure is not yet fully understood, rendering their evaluation and prognosis challenging before treatment (35). An artificial intelligence (AI) algorithm was presented by Markus Lachmann at TCT 2023, which enables the reliable prediction of pulmonary artery pressures, typically measured by right-heart catheterization, using echocardiographic parameters. This advancement allows for the non-invasive assessment of RV-PA coupling to predict survival rates following TTVI since PA pressures might be underestimated in severe TR with echocardiography (36). In the future, similar AI algorithms and models could be employed for the prognostic evaluation of patients prior to intervention (37).

### 5.5 Theoretical considerations of the treatment strategies on RV function

The reduction of TR is accompanied by changes in RV hemodynamics and function, leading to RV reverse remodeling. However, the mechanisms underlying this remodeling vary. Replacing the diseased tricuspid valve with TTVR eliminates TR and consequently affects RV function. Data from the TRISCEND trial showed significant decreases in RV dimensions and FAC, which are consistent with expected changes according to pressure-volume diagrams. TAPSE was significantly reduced at follow-up (11), but this measurement cannot be adequately utilized as the prosthesis is anchored within the annulus, limiting its movement capability. Changes in the RV function observed following heterotopic valve implantation are attributed to similar mechanisms as TTVR. However, these treatment modalities do not address the underlying cause of TR, typically secondary to enlargement of the annular dimensions. T-TEER primarily reduces the severity of TR, and a reduction of the tricuspid annulus following T-TEER was observed, reflecting a certain proportion of an annuloplasty effect (38). Direct annuloplasty, which mimics established surgical repair, seems to be the most physiological treatment option and showed a strong reverse-remodeling in a magnetic resonance imaging-based study (39). Whether this translates to clinical benefit, however, remains to be investigated.

### 5.6 What developments are expected?

The field of TTVI is rapidly evolving, with clinical experience growing daily. As we await further research into the potential prognostic benefits for specific patient groups, preliminary data suggest that patients may benefit from early intervention (36). However, this hypothesis requires validation in future trials. Expected insights from ongoing prospective trials include follow-up data from the PASTE registry for the PASCAL system for TR, as well as long-term follow-up from TRISCEND, TRILUMINATE, and TRILUMINATE Pivotal trials. The latter is especially of interest as it may reveal a prognostic benefit of T-TEER over OMT alone over time. In selecting the appropriate treatment modality and timing, it is crucial to consider health status and quality-of-life assessments. While the impact of RV dysfunction and cardiac remodeling on therapeutic success and prognosis is undeniable, our understanding of these factors remains incomplete. Artificial intelligence algorithms could play a crucial role in processing echocardiographic data to predict RV failure post-TTVI (even non-linear associations). While, to our knowledge, no prospective trials are ongoing in this regard, artificial intelligence assessment of cardiac imaging modalities, especially computed tomography, might prove imperative in the years to come. As device technology advances and new devices enter the market, our portfolio of treatment options will expand, enabling more tailored treatment choices based on individual anatomical characteristics and clinical risk profiles. Additionally, combined approaches utilizing different treatment modalities may be increasingly adopted.

## 6 Conclusion

TTVIs have proven to be safe and efficient, representing a significant advancement in TR treatment. Although no clear prognostic benefits in terms of survival or rehospitalization rates have been established, these interventions are consistently associated with notable improvements in patients’ health status and quality of life, which in fact might be more important in the so far elderly populations. Additionally, recent data emphasize the importance of early evaluation and treatment, which correlate with better clinical outcomes. The adequate assessment of RV function is challenging but critical to patient outcomes. This highlights an urgent need for improved diagnostic and management strategies. Further research is essential to gain more insights into the role of RV function and remodeling, and their respective impacts on quality of life and long-term survival. Ultimately, this knowledge will lead to optimized treatment protocols for patients suffering from TR.

## References

[1] Vahanian A Beyersdorf F Praz F Milojevic M Baldus S Bauersachs J 2021 ESC/EACTS guidelines for the management of valvular heart disease. Eur Heart J. (2022) 43:561–632. 10.1093/eurheartj/ehab395 34453165

[2] Topilsky Y Maltais S Medina Inojosa J Oguz D Michelena H Maalouf J Burden of tricuspid regurgitation in patients diagnosed in the community setting. JACC Cardiovasc Imaging. (2019) 12:433–42. 10.1016/j.jcmg.2018.06.014 30121261

[3] Benfari G Antoine C Miller WL Thapa P Topilsky Y Rossi A Excess mortality associated with functional tricuspid regurgitation complicating heart failure with reduced ejection fraction. Circulation. (2019) 140:196–206. 10.1161/CIRCULATIONAHA.118.038946 31117814

[4] Bannehr M Roland Edlinger C Kahn U Liebchen J Okamoto M Hähnel V Open access natural course of tricuspid regurgitation and prognostic implications. Heart. (2021) 8:1529. 10.1136/openhrt-2020-001529 33563777 PMC7875290

[5] Welle GA Hahn RT Lindenfeld J Lin G Nkomo VT Hausleiter J New approaches to assessment and management of tricuspid regurgitation before intervention. JACC Cardiovasc Interv. (2024) 17:837–58. 10.1016/j.jcin.2024.02.034 38599687

[6] Lurz P von Bardeleben R S Weber M Sitges M Sorajja P Hausleiter J Transcatheter edge-to-edge repair for treatment of tricuspid regurgitation. J Am Coll Cardiol. (2021) 77:229–39. 10.1016/j.jacc.2020.11.038 33478646

[7] Prihadi EA Delgado V Leon MB Enriquez-Sarano M Topilsky Y Bax JJ. Morphologic types of tricuspid regurgitation: characteristics and prognostic implications. JACC Cardiovasc Imaging. (2019) 12:491–9. 10.1016/j.jcmg.2018.09.027 30846123

[8] Otto CM Nishimura RA Bonow RO Carabello BA rwin JP Gentile F 2020 ACC/AHA guideline for the management of patients with valvular heart disease: executive summary: a report of the American College of Cardiology/American Heart Association joint committee on clinical practice guidelines. Circulation. (2021) 143:E35–71. 10.1161/CIR.0000000000000932 33332149

[9] Sorajja P Whisenant B Hamid N Naik H Makkar R Tadros P Transcatheter repair for patients with tricuspid regurgitation. N Engl J Med. (2023) 388:1833–42. 10.1056/NEJMoa2300525 36876753

[10] Gray WA Abramson SV Lim S Fowler D Smith RL Grayburn PA 1-year outcomes of cardioband tricuspid valve reconstruction system early feasibility study. JACC Cardiovasc Interv. (2022) 15:1921–32. 10.1016/j.jcin.2022.07.006 36202561

[11] Makkar R. TRISCEND study 2-year outcomes: transfemoral transcatheter tricuspid valve replacement. In: PCR London Valves 2023: Tricuspid Late-Breaking Trials; 19–21 Nov 2023. London (2023).

[12] Lurz P. TRISCEND II: a randomised trial of transcatheter tricuspid valve replacement vs medical therapy. In: PCR London Valves 2023: The Top Late-Breaking Trials; 19–21 Nov 2023. London (2023).

[13] Zhang Y Lu F Li W Chen S Li M Zhang X A first-in-human study of transjugular transcatheter tricuspid valve replacement with the LuX-valve plus system. EuroIntervention. (2023) 18:e1088–9. 10.4244/EIJ-D-22-00517 36062995 PMC9909447

[14] Lu FL Ma Y An Z Cai CL Li BL Song ZG First-in-man experience of transcatheter tricuspid valve replacement with LuX-valve in high-risk tricuspid regurgitation patients. JACC Cardiovasc Interv. (2020) 13:1614–6. 10.1016/j.jcin.2020.03.026 32646711

[15] Dreyfus J. TTVR with the LuX valve system pivotal trial: one-year outcomes from a multicenter experience. In: PCR London Valves 2023: The Top Late-Breaking Trials; 19–21 Nov 2023. London (2023).

[16] Stocker TJ. Last, but not least: TricValve implantation reduces heart failure burden in severe tricuspid regurgitation. JACC Cardiovasc Interv. (2024) 17:73–5. 10.1016/j.jcin.2023.11.002 38069987

[17] D’Amario D Rodolico D Delvinioti A Laborante R Iacomini C Masciocchi C Eligibility for the 4 pharmacological pillars in heart failure with reduced ejection fraction at discharge. J Am Heart Assoc. (2023) 12:e029071. 10.1161/JAHA.122.029071 37382176 PMC10356099

[18] Solomon SD McMurray JJV Claggett B de Boer RA DeMets D Hernandez AF Dapagliflozin in heart failure with mildly reduced or preserved ejection fraction. N Engl J Med. (2022) 387:1089–98. 10.1056/nejmoa2206286 36027570

[19] Zannad F Ferreira JP Pocock SJ Anker SD Butler J Filippatos G SGLT2 inhibitors in patients with heart failure with reduced ejection fraction: a meta-analysis of the EMPEROR-reduced and DAPA-HF trials. Lancet. (2020) 396:819–29. 10.1016/S0140-6736(20)31824-9 32877652

[20] Spertus J Peterson E Conard MW Heidenreich PA Krumholz HM Jones P Monitoring clinical changes in patients with heart failure: a comparison of methods. Am Heart J. (2005) 150:707–15. 10.1016/j.ahj.2004.12.010 16209970

[21] Green CP Porter CB Bresnahan DR Spertus JA. Development and evaluation of the Kansas city cardiomyopathy questionnaire: a new health Status measure for heart failure. J Am Coll Cardiol. (2000) 35:1245–55. 10.1016/s0735-1097(00)00531-3 10758967

[22] Nickenig G. Percutaneous dge-to-edge repair for tricuspid regurgitation: 3-year outcomes from the TRILIMUNATE trial. In: PCR London Valves 2023: The Late-Breaking Trials; 19–21 Nov 2023. London (2023).

[23] Estevez-Loureiro R. Real-world outcomes for tricuspid edge-to-edge repair: initial 2-year outcomes from the BRIGHT trial. In: PCR London Valves 2023: Tricuspid Late-Breaking Trials; 19–21 Nov 2023. London (2023).

[24] Kodali SK Hahn RT Davidson CJ Narang A Greenbaum A Gleason P 1-year outcomes of transcatheter tricuspid valve repair. J Am Coll Cardiol. (2023) 81:1766–76. 10.1016/j.jacc.2023.02.049 37137586

[25] Dannenberg V Bartko PE Andreas M Bartunek A Goncharov A Gerçek M Tricuspid edge-to-edge repair for tricuspid valve prolapse and flail leaflet: feasibility in comparison to patients with secondary tricuspid regurgitation. Eur Heart J Cardiovasc Imaging. (2024) 25:365–72. 10.1093/ehjci/jead264 37861385 PMC10883724

[26] Arnold S V Goates S Sorajja P Adams DH von Bardeleben RS Kapadia SR Health status after transcatheter tricuspid-valve repair in patients with severe tricuspid regurgitation. J Am Coll Cardiol. (2024) 83:1–13. 10.1016/j.jacc.2023.10.008 37898329

[27] Blasco-Turrión S Briedis K Estévez-Loureiro R Sánchez-Recalde A Cruz-González I Pascual I Bicaval TricValve implantation in patients with severe symptomatic tricuspid regurgitation: 1-year follow-up outcomes. JACC Cardiovasc Interv. (2024) 17:60–72. 10.1016/j.jcin.2023.10.043 38069986

[28] Ochs L Körber MI Wienemann H Tichelbäcker T Iliadis C Metze C Comparison of transcatheter leaflet-approximation and direct annuloplasty in tricuspid regurgitation. Clin Res Cardiol. (2024) 113:126–37. 10.1007/s00392-023-02287-0 37642720 PMC10808287

[29] Dreyfus J Audureau E Bohbot Y Coisne A Lavie-Badie Y Bouchery M TRI-SCORE: a new risk score for in-hospital mortality prediction after isolated tricuspid valve surgery. Eur Heart J. (2022) 43:654–62. 10.1093/eurheartj/ehab679 34586392 PMC8843795

[30] Dreyfus J Galloo X Taramasso M Heitzinger G Benfari G Kresoja K-P TRI-SCORE and benefit of intervention in patients with severe tricuspid regurgitation. Eur Heart J. (2024) 45:586–97. 10.1093/eurheartj/ehad585 37624856

[31] Gerçek M Narang A Körber MI Friedrichs KP Puthumana JJ Ivannikova M GLIDE score A scoring system for prediction of procedural success in tricuspid valve transcatheter edge-to-edge repair. JACC Cardiovasc Imaging. (2024) 17:729–42. 10.1016/j.jcmg.2024.04.008 38842961

[32] Stolz L Doldi PM Kresoja KP Bombace S Koell B Kassar M Applying the TRILUMINATE eligibility criteria to real-world patients receiving tricuspid valve transcatheter edge-to-edge repair. JACC Cardiovasc Interv. (2024) 17:535–48. 10.1016/j.jcin.2023.11.014 37987997

[33] Bashore TM Serfas JD. Isolated tricuspid valve surgery. J Am Coll Cardiol. (2017) 70:2961–3. 10.1016/j.jacc.2017.10.038 29241484

[34] Brener MI Masoumi A Ng VG Tello K Bastos MB Cornwell WK III Invasive right ventricular pressure-volume analysis: basic principles, clinical applications, and practical recommendations. Circ Heart Fail. (2022) 15:e009101. 10.1161/CIRCHEARTFAILURE.121.009101 34963308 PMC8766922

[35] Rommel KP Taramasso M Ludwig S Bonnet G Thiele H Leon MB Low-cardiac output syndrome after tricuspid valve repair: insights from the TriValve registry. JACC Cardiovasc Interv. (2023) 16:1703–5. 10.1016/j.jcin.2023.05.010 37294229

[36] Gerçek M Goncharov A Narang A Körber M Friedrichs KP Baldridge AS Characterization of screen failures among patients evaluated for transcatheter tricuspid valve repair (TriSelect-study). JACC Cardiovasc Interv. (2023) 16:1579–89. 10.1016/j.jcin.2023.03.036 37438025

[37] Lachmann M. Artificial intelligence-enabled assessment of right ventricular to pulmonary artery coupling refines mortality prediction in patients with severe tricuspid regurgitation undergoing transcatheter tricuspid valve intervention. In: TCT 2023; 23–26 Oct 2023. San Francisco, CA (2023).

[38] Russo G Hahn RT Alessandrini H Andreas M Badano LP Braun D Effects of tricuspid transcatheter edge-to-edge repair on tricuspid annulus diameter—data from the TriValve registry. Int J Cardiol. (2024) 405:131934. 10.1016/j.ijcard.2024.131934 38437953

[39] Gerçek M Roder F Friedrichs KP Ivannikova M Goncharov A Fortmeier V Right heart remodeling assessed by cardiac magnetic resonance imaging following transcatheter tricuspid valve annuloplasty. JACC Cardiovasc Imaging. (2023) 16:862–3. 10.1016/j.jcmg.2022.12.014 36881419

